# The effects of hibernation and forced disuse (neurectomy) on bone properties in arctic ground squirrels

**DOI:** 10.14814/phy2.12771

**Published:** 2016-05-24

**Authors:** Lori K. Bogren, Erin L. Johnston, Zeinab Barati, Paula A. Martin, Samantha J. Wojda, Ian G. Van Tets, Adrian D. LeBlanc, Seth W. Donahue, Kelly L. Drew

**Affiliations:** ^1^ Chemistry and Biochemistry DepartmentInstitute of Arctic BiologyUniversity of Alaska FairbanksFairbanksAlaska; ^2^ Mechanical Engineering DepartmentColorado State UniversityFort CollinsColorado; ^3^ Department of Biological SciencesUniversity of Alaska AnchorageAnchorageAlaska; ^4^ Universities Space Research AssociationHoustonTexas

**Keywords:** Bone density, disuse, hibernation, muscle atrophy, torpor

## Abstract

Bone loss is a well‐known medical consequence of disuse such as in long‐term space flight. Immobilization in many animals mimics the effects of space flight on bone mineral density. Decreases in metabolism are also thought to contribute to a loss of skeletal mass. Hibernating mammals provide a natural model of disuse and metabolic suppression. Hibernating ground squirrels have been shown to maintain bone strength despite long periods of disuse and decreased metabolism during torpor. This study examined if the lack of bone loss during torpor was a result of the decrease in metabolic rate during torpor or an evolutionary change in these animals affording protection against disuse. We delineated changes in bone density during natural disuse (torpor) and forced disuse (sciatic neurectomy) in the hind limbs of the arctic ground squirrel (AGS) over an entire year. We hypothesized that the animals would be resistant to bone loss due to immobilization and disuse during the winter hibernation season when metabolism is depressed but not the summer active season. This hypothesis was not supported. The animals maintained bone density (dual‐energy X‐ray absorptiometry) and most bone structural and mechanical properties in both seasons. This was observed in both natural and forced disuse, regardless of the known metabolic rate increase during the summer. However, trabecular bone volume fraction (microcomputed tomography) in the distal femur was lower in neurectomized AGS at the study endpoint. These results demonstrate a need to better understand the relationship between skeletal load (use) and bone density that may lead to therapeutics or strategies to maintain bone density in disuse conditions.

## Introduction

Long‐term space flight produces a decrease in bone density that poses a hazard for astronauts during and after missions in space (LeBlanc et al. 2000; Sibonga et al. 2007). Bone loss is due in part to disuse as long‐term bed rest mimics the effects of space flight on bone mineral density (BMD; Spector et al. 2009). In rats, disuse due to sciatic neurectomy induces a decrease in BMD 8 weeks post neurectomy compared to an increase in BMD in sham animals (Kuwamoto et al. 2007). Similarly, in humans, denervation from spinal cord injury causes up to a 1% decrease in BMD per week during the initial months after injury followed by a slower loss of bone mass (Bauman and Cardozo 2015). These studies show unbalanced bone remodeling, which is the hallmark of disuse and space flight leading to bone loss (Weinreb et al. 1989; Li et al. 2005; McGee‐Lawrence et al. 2008; Leblanc et al. 2013). Exactly how skeletal load (use), metabolism and energy homeostasis, influence bone density remains unknown.

One approach in biomedical research is to examine naturally occurring adaptations in animals and then mimic these mechanisms for therapeutic purposes (Carey et al. 2012). August Krogh proposed that some organisms are particularly well‐suited for studying specific problems that affect humans, a principle now known as Krogh's principle (Krogh 1929). Hibernating mammals are known to resist bone loss during prolonged periods of disuse associated with dormancy and thus may hold clues about how to preserve bone during disuse in humans.

Previous studies have found that small hibernating mammals maintain bone density (Utz et al. 2009; McGee‐Lawrence et al. 2011; Doherty et al. 2012; Wojda et al. 2012) and mechanical properties (Utz et al. 2009; McGee‐Lawrence et al. 2011; Wojda et al. 2012) during the hibernation season. However, studies have not been conducted to ascertain the ability of hibernating animals to maintain bone mass and strength during extended periods of imposed disuse in both the winter and summer seasons. Determining if bone density and strength are maintained with imposed disuse during both the winter hibernation and summer active seasons will delineate if the observed maintenance of bone density in small hibernators during the hibernation season is due to seasonal, biochemical changes (e.g., reduced metabolism) or is a species phenotype that persists regardless of seasonal influences.

Here, we sought to delineate changes in bone density that occur during natural disuse (torpor) and forced disuse (sciatic neurectomy) in the hind limbs of the arctic ground squirrel (*Urocitellus parryii*, AGS) over the winter and summer seasons. In addition, we determined changes in mechanical strength and trabecular bone properties after a year of imposed disuse. We tested the hypothesis that the AGS will resist bone loss due to limb immobilization during the winter, but not the summer season and that overall mechanical weakening of the bone will occur with prolonged disuse. A total of nine animals were used with right leg operated (neurectomy) or sham‐operated and with left legs serving as contralateral controls. Results failed to support our hypothesis. We found that AGS maintained total bone density and strength with both natural and forced disuse and that this persisted throughout both the winter hibernation and the summer active seasons. Understanding how hibernating mammals maintain bone density with chronic disuse may lead to therapeutics or strategies to maintain bone density in space, prolonged immobilization, or in other scenarios of extended disuse.

## Materials and Methods

### Animals and ethics statement

All animal procedures were performed in strict accordance with the Guide for the Care and Use of Laboratory Animals and approved by the Animal Use and Care Committee of the University of Alaska Fairbanks. Both male and female AGS (474–821 g, *n* = 9) were used in this study due to the availability of wild‐caught animals. Capture and holding of AGS was performed under permit by the Alaska Department of Fish and Game. Adult AGS were live trapped north of the Brooks Range (66°38′N, 149°38′W) in Alaska during July immediately prior to the experiments. Animals were housed in 12″×19″×12″ cages at 22°C under light conditions based on 69° latitude from time of capture until late August when they were then transferred to a cold chamber (2°C) with 4:20 light:dark for the duration of the experiment (November–October). Food and water were provided ad libitum. Summer euthermic status of an animal was assessed by body temperature, activity, and lack of spontaneous torpor for at least 4 weeks. Animals were considered to be in the winter hibernation season when they had been having regular spontaneous torpor bouts. Torpor bouts last for 1–3 weeks (Twente and Twente 1965) and are broken up by interbout arousals (IBA) lasting ~12–20 h (Karpovich et al. 2009) where body temperature is returned to euthermic ranges and some minimal physical activity from shifting position or moving in the housing can occur. During such periods, mechanical forces generated by physical activity may be exerted on the bones via muscles contractions (Klein‐Nulend et al. 2012).

### Limb immobilization

Adult AGS (*n* = 5, 2 male and 3 female) had their right hind limb immobilized via sciatic neurectomy in October, 2 months after capture and at the onset of the hibernation season. Animals were aged based on size and tooth wear. All animals used in this study were defined as adults. Under anesthesia (isoflurane, induced at 5%, maintained at 2.5–3% via inhalation mixed with 100% medical grade oxygen), a curvilinear incision starting at greater trochanter and ending proximal to the stifle was made and the sciatic nerves exposed. In the neurectomy animals (NEUR), in the right hind limb (test) a 0.5 cm section of the nerve was excised. The muscle and skin were sutured. As a control, sham neurectomies (SHAM) were performed on adult AGS (*n* = 4, 1 male and 3 female) where the animals underwent the surgical procedure, except nerve transection, on the right hind limb (test). Left hind limbs of both groups were untreated (control). Buprenorphine (0.05 mg/kg) was administered immediately prior to surgery and every twelve hours afterwards for 2 days. Directly after surgery, animals were observed until ambulatory. Thereafter, animals were monitored daily during the recovery period for complications or incision site mutilation. All incision sites closed within 12 days. Subsequent analysis was conducted in a blinded fashion without knowledge of treatment group.

Animals had each hind limb tested for immobility prior to the last dual‐energy X‐ray absorptiometry (DXA) measurement at the end of the experiment by placing the front quarters of each animal in a slightly suspended tube. A solid object was then placed under either the right or left hind paw. The ability of the animal to feel and use the object to push its hind quarters into the tube with the rest of its body was then recorded on a numerical scale with 1 indicating no movement or use of the hind limb, 3 indicating the paw did intentionally rest on the object but that there was no ability for the limb to push the animal upwards, and 5 indicating that the animal was able to place the paw on the object and use the hind limb to push its hind quarters into the tube. Both hind limbs were tested three times for each animal and the average recorded. Additionally, each hind paw was pinched and the animal's ability to feel the pinch and retract its paw recorded. None of the limbs in which the sciatic nerve had been severed responded to pinch sensation and could not be used for locomotion. However, both limbs of the SHAM animals and the control limb of the NEUR animals were able to detect the pinch and had full mobility function. NEUR animals also showed evidence of dragging their immobilized limb (fur rubbed off on the top of the foot).

### Dual‐energy X‐ray absorptiometry

**Figure 1:**
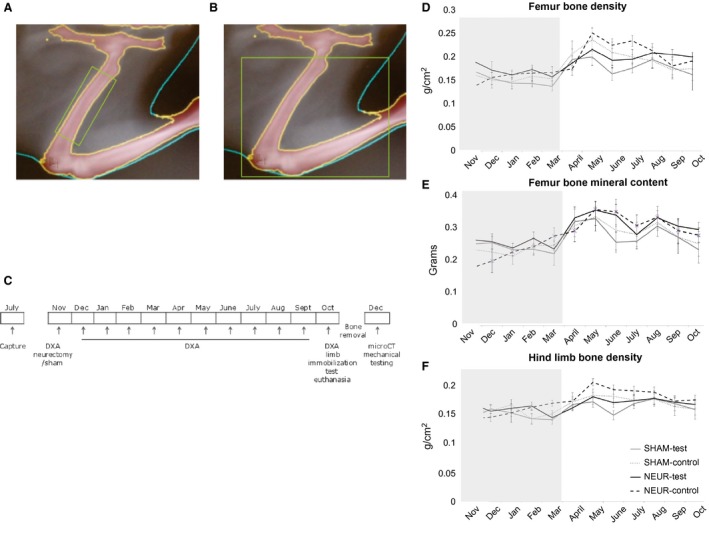
Dual‐energy X‐ray absorptiometry (DXA) bone density and mineral content are maintained during the hibernation and increase during active seasons. (A) Femur region of interest (ROI). (B) Hind limb ROI. (C) Timeline of capture, surgery, DXA measurements, *μ* CT, and mechanical testing. Animals were captured from the wild in July, surgeries and baseline DXA measurement were obtained in early November. DXA scans though September were conducted the first week of each month. In October, the final DXA measurements were obtained and animals were euthanized. Bones were removed, cleaned of nonosseous tissue between October and December. *μ* CT and mechanical testing were conducted in December. Femurs from all test limbs showed similar trends of maintaining bone density (D) and mineral content (E) during the hibernation season and increasing at the start of the active season. Total hind limb bone density (F) also was maintained during hibernation and increased in the spring. Control indicates left hind limb that did not undergo any procedure. Test is the right hind limb that had sciatic neurectomy (NEUR,* n* = 5) or sham surgery (SHAM,* n* = 4). All data are mean ± SEM. Gray area denotes hibernation season during winter 2013. Statistical analysis via ANOVA followed by Tukey's post hoc test. Significance was determined at *P* ≤ 0.05.

Bone density, lean tissue, and percentage of fat tissue in both the immobilized or sham leg (test, right) and the nonimmobilized leg (control, left) were monitored noninvasively via DXA (PIXImus 2, Lunar/GE; Madison, WI; Stevenson and van Tets 2008). This was done on a monthly basis over the course of a year (femur and limb regions of interest shown in Fig. 1A and B) beginning in November at the start of the hibernation season. Nontorpid animals were scanned under anesthesia as previously described. At the end of the year, animals were euthanized via decapitation under a surgical plane of isoflurane anesthesia and the femurs extracted for further analysis via microcComputed tomography (*μ*CT) and mechanical testing (three‐point bending). The experimental timeline is shown in Figure 1C.

### Microcomputed tomography

The left and right femurs from each animal were stored wrapped in saline‐soaked gauze at −20°C. Trabecular bone properties were assessed by *μ*CT. The distal femur was scanned at 70 kVp and 114 *μ*A with a uCT80 system (Scanco Medica; Wayne, PA) at 10 *μ*m resolution. To ensure the same relative location was scanned in all bones the scan and evaluation regions were scaled to bone length. Scans started at a distance of 0.92–1.03 mm proximal to the distal femoral growth plate (the start of the scan region was equal to a distance of 2% of the bone length) and the length of the evaluated region was equal to 5% of total bone length (2.28–2.56 mm). Measurements of interest included trabecular bone volume fraction (BV/TV), trabecular tissue mineral density (Tb.M.Dn, mgHA/cm^3^), trabecular thickness (Tb.Th, mm), trabecular number (Tb.N, 1/mm), and trabecular separation (Tb.Sp, mm).

### Mechanical testing

The right and left femurs of each animal were thawed and rehydrated in 0.9% (0.15 mol/L) saline solution for approximately four hours prior to mechanical testing. Each femur was loaded to failure in three‐point bending with the anterior side in tension. Tests were performed on a MTS mechanical testing system (Eden Prairie, MN) with a crosshead speed of 1 mm/min and data sampling rate of 1 kHz. Due to the size and geometry of the bones, a small preload (1–10 N) was applied to ensure the bone did not rotate during loading. Average span for testing was 24.3 mm.

Ultimate force (N) and failure energy (J) were calculated from the load‐deformation data obtained during three‐point bending (McGee‐Lawrence et al. 2011). Ultimate force was determined by the maximum load achieved during testing. Failure energy was calculated as the area under the curve up to the point of fracture. Modulus of toughness (*u*) and ultimate stress were calculated as previously described (Wojda et al. 2012).

### Geometrical properties

Cortical area (Ct.Ar) and maximum moment of inertia (*I*_max_) were calculated with image analysis software (Scion Corporation, Frederick, MD) from images of midshaft femur cross sections obtained via a digital camera (SPOT Insight QE, Diagnostic Instruments, Sterling Heights, MI) with a Nikon lens. To remove the effect of variations in animal size on cross‐sectional parameters, these parameters were normalized by (femur length)^4^ for *I*_max_ and (femur length)^2^ for Ct.Ar (Casinos and Viladiu 1993; Heinrich and Biknevicius 1998).

### Statistics

A priori power analysis was performed (using G*Power software, version 3.1.7; Universität Kiel, Kiel, Germany) to minimize the likelihood of Type II statistical errors. No dataset from a similar study was available for the exact calculation of power appropriate for the ANOVA design used in this study. Therefore, we powered this study to reflect a medium to large effect size (partial eta squared: 0.06 [medium effect] − 0.14 [large effect]) for the variable of apparent density of the femur based on previously published research in rats and golden mantled ground squirrels (Kuwamoto et al. 2007; Utz et al. 2009). The predicted effect size (partial *η* ^2^ = 0.1) was then used for a repeated measures ANOVA design with within and between factors interaction. A total sample size of 8 was calculated; however, the subsequent sample size exceeded this estimate.

Data are presented as mean ± SEM. All data were tested for normality and found to be normally distributed according to Kolmogorov–Smirnov and Shapiro–Wilk tests prior to further statistical analysis. Mass at start of hibernation, number of spontaneous torpor bouts, and average length of torpor bouts (days) were statistically analyzed via student's *t*‐test. DXA measurements were analyzed via three‐way ANOVA (treatment [SHAM or NEUR] × limb [control‐left hind limb or test‐right hind limb] × time [month]) with two repeated measures of time and limb. The *μ*CT and mechanical test data were analyzed by two‐way ANOVA (treatment × limb) with repeated measure of limb. To assess the effect of season, differences in DXA parameters during the hibernation season (November–March), at the start of the active season (March–May), at the end of the active season (June–September) and over the entire year (November–October) were analyzed via two‐way ANOVA (treatment × limb) with a repeated measure of limb. Difference was calculated as final value − initial value. All significant effects in ANOVAs were followed by a Tukey's post hoc test or *t*‐*test*s. Statistical significance was considered to be a *P*‐value of <0.05. A summary table of the statistical finding is presented in Table 1. All statistical analyses were performed in IBM Statistics, Version 22.0 (Armonk, NY).

**Table 1:** Summary statistics table

Variable	ANOVA test	*F*‐value (df)	*P*‐value
Total body mass	Time × treatment	*F*(11, 77) = 0.791	0.648
	Time	*F*(11, 77) = 1.875	0.056
BMD‐whole limb	Time × limb × treatment	*F*(11, 77) = 1.292	0.245
	Time	*F*(11, 77) = 7.065	<0.001
	Limb × time	*F*(11, 77) = 2.738	0.005
BMD‐whole limb – body mass norm	Time × limb × treatment	*F*(11, 77) = 1.201	0.301
	Time	*F*(11, 77) = 3.183	0.001
	Limb × time	*F*(11, 77) = 2.693	0.006
BMD‐femur	Time × limb × treatment	*F*(11, 77) = 0.292	0.986
	Time	*F*(11, 77) = 6.102	<0.001
BMD‐femur – body mass norm	Time × limb × treatment	*F*(11, 77) = 0.234	0.994
	Time	*F*(11, 77) = 4.847	<0.001
BMC femur	Time × limb × treatment	*F*(11, 77) = 0.668	0.764
	Time	*F*(11, 77) = 7.013	<0.001
	Limb × time	*F*(11, 77) = 1.957	0.045
BMC femur – body mass norm	Time × limb × treatment	*F*(11, 77) = 0.703	0.732
	Time	*F*(11, 77) = 4.589	<0.001
	Limb × time	*F*(11, 77) = 2.037	0.036
Total lean tissue	Time × limb × treatment	*F*(11, 77) = 1.763	0.075
	Time	*F*(11, 77) = 16.719	<0.001
Total lean tissue – body mass norm	Time × limb × treatment	*F*(11, 77) = 1.980	**0.042**
	Time	*F*(11, 77) = 34.717	<0.001
Total fat tissue	Time × limb × treatment	*F*(11, 77) = 0.497	0.899
	Time	*F*(11, 77) = 14.125	<0.001
Total fat tissue – body mass norm	Time × limb × treatment	*F*(11, 77) = 0.470	0.916
	Time	*F*(11, 77) = 17.873	<0.001
Fat percent	Time × limb × treatment	*F*(11, 77) = 0.681	0.752
	Time	*F*(11, 77) = 28.724	<0.001
	Limb × time	*F*(11, 77) = 2.775	0.004
Fat percent – body mass norm	Time × limb × treatment	*F*(11, 77) = 0630	0.798
	Time	*F*(11, 77) = 15.012	<0.001
	Limb × time	*F*(11, 77) = 2.805	0.004
Total lean tissue: November–October	Limb × treatment	*F*(1, 7) = 5.204	0.057
	Limb		0.175
Total lean tissue: March–May	Limb × treatment	*F*(1, 7) = 0.057	0.817
	Limb		0.704
Total lean tissue: November–March	Limb × treatment	*F*(1, 7) = 14.792	**0.006**
	Limb		0.979
Total lean tissue: May–September	Limb × treatment	*F*(1, 7) = 0.254	0.630
	Limb		0.924
Fat percent: November–October	Limb × treatment	*F*(1, 7) = 0.343	0.577
	Limb		0.333
Fat percent: March–May	Limb × treatment	*F*(1, 7) = 1.775	0.225
	Limb		0.497
Fat percent: November–March	Limb × treatment	*F*(1, 7) = 0.944	0.364
	Limb		0.626
Fat percent: May–September	Limb × treatment	*F*(1, 7) = 0.473	0.514
	Limb	*F*(1, 7) = 6.606	0.037
Femur BMD: November–October	Limb × treatment	*F*(1, 7) = 0.264	0.623
	Limb	*F*(1, 7) = 6.766	0.035
Femur BMD: March–May	Limb × treatment	*F*(1, 7) = 1.999	0.200
	Limb	*F*(1, 7) = 7.909	0.026
Femur BMD: November–March	Limb × treatment	*F*(1, 7) = 0.007	0.938
	Limb	*F*(1, 7) = 8.800	0.021
Femur BMC: November–October	Limb × treatment	*F*(1, 7) = 1.130	0.323
	Limb	*F*(1, 7) = 4.194	0.080
Femur BMC: March–May	Limb × treatment	*F*(1, 7) = 0.024	0.881
	Limb		0.251
Femur BMC: November–March	Limb × treatment	*F*(1, 7) = 0.068	0.802
	Limb	*F*(1, 7) = 10.689	0.014
UI stress	Limb × treatment	*F*(1, 7) = 1.976	0.203
Mod T	Limb × treatment	*F*(1, 7) = 2.301	0.173
*I*_max_ (absolute value of log10 function)	Limb × treatment	*F*(1, 7) = 1.156	0.318
C area	Limb × treatment	*F*(1, 7) = 0.759	0.412
Bone volume/total volume	Limb × treatment	*F*(1, 7) = 15.224	**0.006**
	Limb	*F*(1, 7) = 51.258	<0.001
Trabecular number	Limb × treatment	*F*(1, 7) = 0.001	0.983
	Limb	*F*(1, 7) = 6.496	0.038
Trabecular thickness	Limb × treatment	*F*(1, 7) = 9.135	**0.019**
	Limb	*F*(1, 7) = 11.721	0.011
Trabecular separation	Limb × treatment	*F*(1, 7) = 0.961	0.360
	Limb	*F*(1, 7) = 5.698	0.048
Material mineral density	Limb × treatment	*F*(1, 7) = 2.108	0.183
	Limb	*F*(1, 7) = 5.048	0.059
Ultimate force	Limb × treatment	*F*(1, 7) = 1.787	0.223
	Limb	*F*(1, 7) = 0.004	0.949
Failure energy	Limb × treatment	*F*(1, 7) = 2.207	0.181
	Limb	*F*(1, 7) = 0.932	0.367

Bold indicates significant value (*P* ≤ 0.05).

## Results

### Dual‐energy X‐ray absorptiometry of bone and tissue

There was no difference found in either bone mineral content (BMC) or BMD between any limb group (NEUR‐test, NEUR‐control, SHAM‐test, SHAM‐control) during the time course for either the femur or total hind limb (BMC and BMD, time × leg × treatment, 0.245 < *P* < 0.986; Fig. 1D–F). The same was found when data were normalized for body mass at time of measurement (time × leg × treatment, 0.301 < *P* < 0.994). All groups maintained femur BMD and BMC throughout the hibernation season (November–March; hibernation data shown in Table 2). At the start of the next hibernation season (October, 2013), the animals had the same BMD and BMC they initially had at the start of the experiment (November, 2012). Between the end of hibernation (March) and the early active season (May), animals in both the NEUR and SHAM groups showed an increase in BMD (main interaction of time × limb, *P* = 0.0426) and BMC (main effect of time, *P* < 0.001) but there was no effect of the neurectomy treatment for either parameter.

**Table 2:** Hibernation characteristics of arctic ground squirrel. No differences between treatment groups were found by *t*‐test for mass at start of hibernation, number of spontaneous torpor bouts, nor average length of torpor bouts

Animal number	12‐99	12‐102	12‐104	12‐105	12‐100	12‐101	12‐108	12‐109	12‐110
Treatment	SHAM	SHAM	SHAM	SHAM	NEUR	NEUR	NEUR	NEUR	NEUR
Age	Adult	Adult	Adult	Adult	Adult	Adult	Adult	Adult	Adult
Sex	Male	Female	Female	Female	Male	Female	Female	Female	Male
Mass (g) at start of hibernation	688	474	540	786	715	821	636	611	821
First day of spontaneous torpor	10/12/12	10/01/12	11/21/12	10/26/12	10/30/12	09/13/12	10/17/12	10/20/12	12/21/12
Last day of spontaneous torpor	03/10/13	03/24/13	03/13/13	03/12/13	03/11/13	03/10/13	02/18/13	03/29/13	01/31/13
No. of spontaneous torpor bouts	11	13	10	7	7	9	10	9	5
Average length of torpor bouts (days)	10.7	9.14	11.6	10.1	9.86	10.4	5.40	14.0	7.20

**Figure 2:**
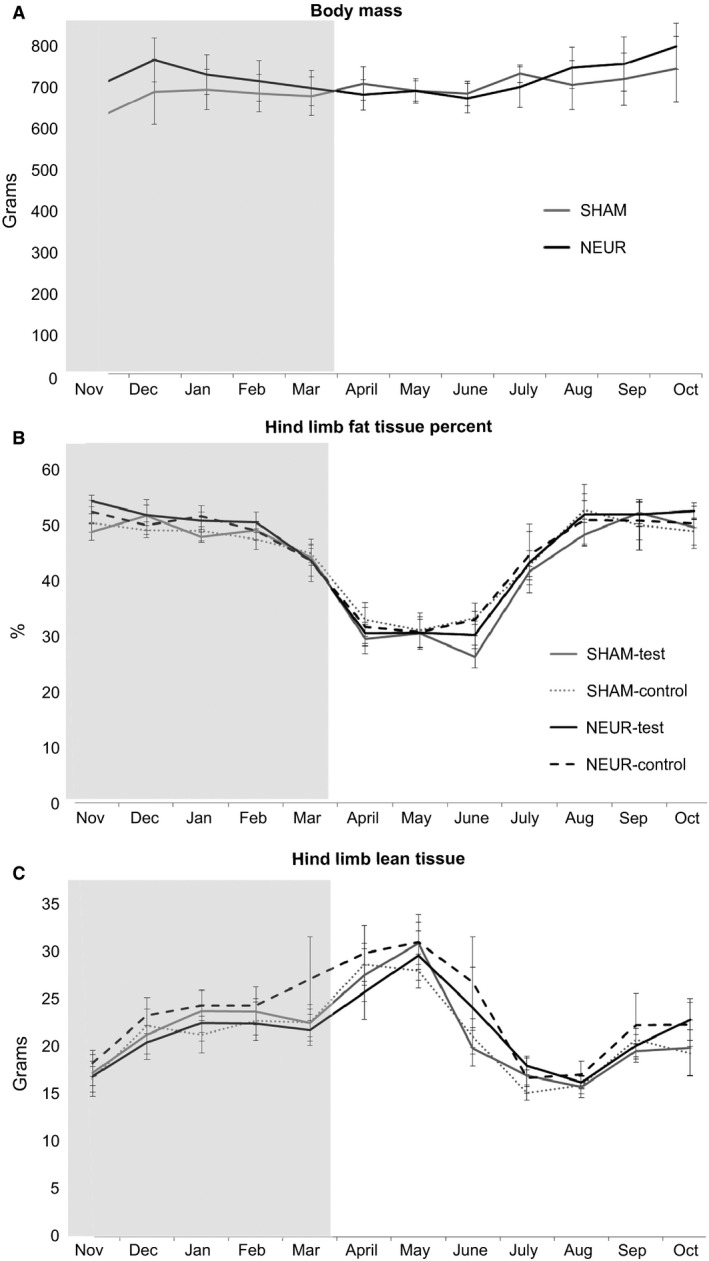
Body mass remained unchanged with or without treatment over the course of the experiments while dual‐energy X‐ray absorptiometry hind limb fat and lean tissue fluctuate between the hibernation and active seasons. Body mass did not differ between test groups (A). Both hind limbs from each test group showed similar trends in dynamics over the hibernation timeline in fat tissue percent (B) and lean tissue mass (C). All data are mean ± SEM. Shaded area indicates the hibernation season during winter 2013. Statistical analysis via ANOVA followed by Tukey's post hoc test. Significance was determined at *P* ≤ 0.05. NEUR *n* = 5 and SHAM *n* = 4.

Neurectomy had no effect on body mass at any time point nor was there a change in body mass in either group over the experiment (Fig. 2A). The percentage of fat and the mass of lean tissue in the total hind limb were measured with DXA (Fig. 2B and C). No differences were found between any of the treatment limbs with respect to either the lean tissue mass or fat tissue percent (time × leg × treatment, *P* = 0.075 and *P* = 0.752, respectively). When normalized for body mass, lean tissue did show a change due to neurectomy (time × leg × treatment, *P* = 0.042) in the month of December (Tukey, *P* = 0.003). The lean tissue increased in the untreated contralateral control limb of the NEUR group in response to treatment during the hibernation season (November–March; leg × treatment, *P* = 0.006) but was not different between groups throughout the early active season (March–May) or summer active season (June–September). Both limbs for each treatment group had no difference in lean mass at the end of the experimental year than at the start (November–October).

### Micro CT of bone structural parameters and three‐point bending for mechanical strength properties

To determine if there were any changes in the structural or mechanical properties of the bone at the study endpoint, the femurs were analyzed with *μ*CT and three‐point bending. AGS retained bone strength, trabecular number and separation regardless of chronic disuse from sciatic neurectomy. There was a significant decrease in trabecular bone volume fraction in the treated limb of the NEUR group (limb × treatment, *P* = 0.006; Table 3) and a significant interaction in the trabecular thickness (limb × treatment, *P* = 0.019) indicating that the effect of treatment on these variables was dependent on treatment limb or contralateral control. However, no difference was found for trabecular thickness within either SHAM or NEUR treatment groups. No differences were found in any of the other parameters tested with *μ*CT or three‐point bending between any of the test limbs (treatment × limb, 0.173 < *P* < 0.983; Table 3).

**Table 3:** *μ*CT and mechanical testing parameters

Parameter	SHAM‐Control (L)	SHAM‐Test (R)	NEUR‐Control (L)	NEUR‐Test (R)	*P*‐value
Trabecular bone volume/total volume	0.087 ± 0.006	0.083 ± 0.007	0.075 ± 0.011^a^	0.059 ± 0.010^b^	0.006
Trabecular number (1/mm)	2.26 ± 0.05	2.22 ± 0.04	1.83 ± 0.16	1.79 ± 0.17	0.983
Trabecular thickness (mm)	0.052 ± 0.004	0.052 ± 0.004	0.064 ± 0.008	0.060 ± 0.008	0.019
Trabecular separation (mm)	0.423 ± 0.009	0.433 ± 0.010	0.548 ± 0.063	0.571 ± 0.074	0.360
Material mineral density (mgHA/ccm)	809 ± 6.34	807 ± 4.67	844 ± 15.3	834 ± 15.3	0.183
Ultimate force (N)	114 ± 11.6	125 ± 20.1	141 ± 13.0	132 ± 9.84	0.223
Failure energy (J)	60.6 ± 16.0	72.6 ± 13.4	122 ± 35.1	65.7 ± 17.9	0.181
Ultimate stress (MPa)	137 ± 12.5	152 ± 10.7	171 ± 20.6	156 ± 14.5	0.203
Modulus of toughness (mJ/mm^3^)	2.93 ± 0.771	3.35 ± 0.730	4.00 ± 1.54	2.62 ± 0.446	0.173
*I*_max_	3.19E‐6 ± 3.04E‐7	2.88E‐6 ± 2.90E‐7	2.68E‐6 ± 2.68E‐7	2.80E‐6 ± 3.62E‐7	0.318
Cortical area	3.17E‐3 ± 1.84E‐4	3.03E‐3 ± 2.30E‐4	3.02E‐3 ± 1.88E‐4	3.14E‐3 ± 2.34E‐4	0.412

Mean values ± SEM, *P*‐value given is for limb × treatment interaction. For significant interactions, different superscript letters indicate difference within treatment group by paired *t*‐test with Bonferroni correction (*P* < 0.05). Cortical area and moment of inertia measures are size‐normalized values where each measure was divided by the appropriate functions of femur length. Size normalization takes into account the differences in bone size between groups (thus the values of these measures in the table are unitless). *n* = 4 for SHAM and *n* = 5 for NEUR.

## Discussion

Here, we show for the first time that AGS maintain bone density and mechanical properties with both natural and forced disuse beyond the hibernation season and into the summer active season. Our DXA findings indicate that the observed maintenance of bone density in the AGS during the hibernation season is not due to seasonal changes (e.g., reduced metabolism) but is due to mechanisms that remain active continuously in this species. These findings are significant because they demonstrate a model of bone density preservation that, when understood, will provide new therapies for disuse bone weakening.

Our findings are consistent with previous studies of bone preservation during hibernation in AGS and other small hibernators (13, 30, 40, 42, 43), but show that in the AGS, BMD and BMC are maintained during extended, non‐hibernation imposed disuse. Overall BMD and BMC were maintained; however, *μ*CT revealed a significant decrease in the trabecular bone volume fraction in both hind limbs of the neurectomized animals. Cortical and trabecular bone cannot be distinguished with DXA nor does it allow for assessment of bone architecture or geometry (Burr and Allen 2013). These parameters were analyzed with *μ*CT. After a year of disuse, AGS had a 29% decrease in trabecular bone volume fraction in the neurectomized limb as compared to the test leg of the sham animals. No other parameters decreased by imposed disuse. In contrast, denervation‐induced disuse in nonhibernating rodents decreases trabecular bone volume fraction, thickness, and number after just 3 weeks by 76%, 54%, and 74%, respectively (Tamaki et al. 2014). Data from *μ*CT data were obtained at the end of the study. As such, it is not clear if this trabecular bone loss occurred in neurectomized animals during hibernation and/or only after they emerged from hibernation. Wodja et al. (2016) have found that trabecular bone volume fraction is greater in hibernating AGS, in contrast to loose trabecular bone structure found during hibernation in adult thirteen‐lined ground squirrels (*Ictidomys tridecemlineatus*; (30)). However, others have found no effect of hibernation on cortical bone quality parameters including bone mass, diameter, volume, apparent density (mass/volume), or whole bone mechanical properties in golden‐mantled ground squirrels (*Spermophilus lateralis*) and thirteen‐lined ground squirrels (Utz et al. 2009; McGee‐Lawrence et al. 2011). Similar results were found in yellow‐bellied marmots (*Marmota flaviventris*; Wojda et al. 2012) and woodchucks (*Marmota monax*; Doherty et al. 2012). While our data are consistent with other studies of bone during the hibernation season, we are the first to show that bone is preserved during the active summer season. In AGS, the ability to maintain bone quality parameters and structural properties during disuse is not dependent on a hibernation phenotype but extends into the summer euthermic state.

Regarding bone mechanical properties, our findings are consistent with previous reports for golden‐mantled ground squirrels (Utz et al. 2009) where the force required to break tibia or femur bones was the same for summer and hibernating groups. In contrast, Utz et al. (2009) found summer golden‐mantled ground squirrels had reduced femur apparent flexural modulus, indicative of a seasonal shift in response to reduced activity. Why we found no change in bone strength and Utz et al., found reduced ability of the femur to withstand bending may be due to species variation. AGS have previously been reported to be resistant to other tissue damage, specifically ischemia/reperfusion injury, in both the summer and hibernation seasons while this resistance is found only during the hibernation season in other ground squirrel species (Frerichs and Hallenbeck 1998; Lindell et al. 2005; Dave et al. 2006; Kurtz et al. 2006; Martin et al. 2008; Jani et al. 2011; Bogren et al., 2014a,b). Further analysis of bone mechanical properties at various time points in the hibernation year may show a seasonal variation in disuse response in the AGS.

Our results demonstrate that in adult ground squirrels, bone properties are largely maintained after sciatic neurectomy regardless of the hibernation season. This differs from previous studies of sciatic neurectomy during hibernation in thirteen‐lined ground squirrels where Zimmerman et al. (1976) found that during the summer months, but not during hibernation, neurectomy led to a loss of bone and muscle mass. Why Zimmerman et al. (1976), found bone and muscle atrophy during disuse in summer while we did not may be due to the exclusive use of juvenile squirrels in the Zimmerman study as well as to difference in techniques used to ascertain the bone and muscle loss. In the Zimmerman findings, bone loss was based on decreases in mineral percent in the bone and increased lacunae size (Zimmerman et al. 1976). In this study, DXA, *μ*CT, and mechanical testing were used to monitor bone changes in the same cohort of animals over the duration and at the end of the study. With these three analyses, differences in bone quality (the sum of physical features and properties that influence bone's ability to resist fracture, not just bone quantity) were measured (Bouxsein 2003).

Changes in nonosseous tissue (lean tissue and fat percent) that occurred with disuse during and after hibernation followed documented trends for obligatory hibernators. Lean tissue (muscle) is maintained or increased during hibernation (Cotton and Harlow 2010; James et al. 2013; Hindle et al. 2015) and fat stores increased dramatically in the latter part of the active season (Dark 2005; Sheriff et al., 2013). Maintenance of lean tissue may be due in part to shivering that occurs in the hibernation season during the process of arousal from torpor (Settnes and Nielsen 1991; Harlow et al. 2004). IBA may also contribute to bone density and lean tissue mass through alterations in metabolism and limited movement in the cages. However, Utz et al. (2009) found that limited movement in cages is not sufficient to mask the negative effects of disuse in summer golden‐mantled ground squirrels. Surprisingly, we did not observe changes in overall body mass as found in free‐living ASG (Buck and Barnes 1999) nor a decrease in hind limb fat percentage over the hibernation season. Body mass maintenance may be due to the captive environment where food is available ad libitum year round and energy expenditure on foraging is eliminated. However, the trend in the fat percent during this study did follow the established pattern: animals have less fat in the summer and more stored fat immediately before the onset of hibernation (Pulawa and Florant 2000; Sheriff et al., 2013).

We conclude that in AGS, bone and tissue are preserved over the course of the hibernation year. We are confident of this conclusion despite limitations to the data collected. One limitation was the unknown age of the animals. With the nature of the model system being analyzed, wild‐caught animals were used so exact age was unknown. Although all of the animals were adults and not in a growth phase, there was no means to discern their exact age. This limitation may have increased variation in the measurements, but would not have biased results. Hibernation patterns also varied among the animals with the beginning of hibernation ranging from September to December and the end of hibernation from January to March, and lasting 41 to 178 days. However, the percentage of days spent torpid during the hibernation period did not differ between groups or affect changes in the bone properties measured. Additionally, we acknowledge that another limitation of this study was lack of access to a dataset from an identical study for exact calculation of a priori power forcing us to use of a qualitative approach to estimate the sample size. Nonetheless, the changes expected from disuse atrophy in other model systems are great enough that a priori power analysis indicated that a total sample size of eight would have a 99.9% power for detecting an effect size of 0.997. Subsequent sample size exceeded this estimate. Moreover, this study now provides data for more precise a priori power calculations for future studies that will be necessary to fully validate the negative findings reported here.

In summary, muscle and bone atrophy during periods of disuse is a significant medical problem for astronauts and those with chronic limited mobility. The remarkable resistance to disuse atrophy shown by hibernating rodents during hibernation, and in AGS throughout the year offers a model for further study of mechanisms of bone preservation that may lead to improved means to protect bone atrophy in human populations.

## Conclusion

Arctic ground squirrel retained most bone properties with both natural and forced disuse and this persisted throughout both the summer active and winter hibernation seasons. In addition, AGS maintained cortical bone mechanical properties regardless of chronic disuse. However, trabecular bone volume fraction and thickness were lower in the neurectomized limbs. These results demonstrate that the maintenance of many bone properties important for resisting bone fracture, such as bone density and mechanical properties, can be preserved during prolonged periods of disuse under reduced and normal metabolic rate in hibernating mammals. Determining the underlying biochemical pathways responsible for the AGS's bone maintenance during imposed disuse could lead to therapies that are not dependent on inducing a hibernation‐like phenotype but could be manipulated pharmacologically in a nonhibernating species to inhibit bone loss that results from prolonged disuse.
